# Global Clustering Quality Coefficient Assessing the Efficiency of PCA Class Assignment

**DOI:** 10.1155/2014/342497

**Published:** 2014-08-26

**Authors:** Mirela Praisler, Stefanut Ciochina

**Affiliations:** Department of Chemistry, Physics and Environment, “Dunarea de Jos” University of Galati, 800008 Galati, Romania

## Abstract

An essential factor influencing the efficiency of the predictive models built with principal component analysis (PCA) is the quality of the data clustering revealed by the score plots. The sensitivity and selectivity of the class assignment are strongly influenced by the relative position of the clusters and by their dispersion. We are proposing a set of indicators inspired from analytical geometry that may be used for an objective quantitative assessment of the data clustering quality as well as a global clustering quality coefficient (GCQC) that is a measure of the overall predictive power of the PCA models. The use of these indicators for evaluating the efficiency of the PCA class assignment is illustrated by a comparative study performed for the identification of the preprocessing function that is generating the most efficient PCA system screening for amphetamines based on their GC-FTIR spectra. The GCQC ranking of the tested feature weights is explained based on estimated density distributions and validated by using quadratic discriminant analysis (QDA).

## 1. Introduction

Invented by Pearson in 1901 [1, 2], principal component analysis (PCA) is still one of the most popular statistical tools, because it helps us inspect the structure of large amounts of data characterizing a given phenomenon. PCA is mostly used in exploratory data analysis in order to identify the clusters formed by samples with similar properties or behavior as well as the most important variables (principal components (PCs)) that explain most of the data variance. Once the natural clustering of the data is known, PCA may be applied to build predictive models that are then used for classification purposes, that is, for assigning the class of unknown samples.

The spreading of computers and user-friendly software applications has allowed in the last decades the use of PCA in a large variety of domains [2]. Useful PCA applications have been recently reported in very different scientific fields, from food science [3] to educational management [4], from economy [5] to environmental research [6], from energy [7] to biometrics [8], and from toxicology [9] to archaeometry [10]. Most of the PCA studies have been performed in analytical chemistry (chemometrical applications), as most of the instruments are currently linked to computers that allow the storage of huge amounts of data. For example, PCA has been used to explore, sort, and group spectral data obtained with techniques such as UV-visible (UV-VIS), near-infrared (NIR) and mid-infrared (MIR) spectroscopy [11], Fourier transform near-infrared spectroscopy (FT-NIR) [12], diffuse reflectance infrared Fourier transform spectroscopy [13], attenuated total reflection Fourier transform infrared spectroscopy (ATR-FTIR) [10], gas chromatography-Fourier transform infrared spectroscopy (GC-FTIR) [9], gas chromatography-mass spectroscopy [14], or inductively coupled plasma optical emission spectrometry (ICP-OES) [15].

In order to operate data and make predictions, PCA is also often used in combination with other methods, such as linear discriminant analysis (LDA) [8, 13], soft independent modeling of class analogy (SIMCA) [9], cluster analysis (CA) [15], or artificial neural networks (ANN) [14, 16]. More specifically, PCA is used to reduce the dimensionality of complex data sets and to reveal sometimes hidden, simplified structures that underlie it. These structures form, in the PCA score plots, clusters that are expressing the data in such a way as to highlight their similarities and differences. In other words, PCA is an* unsupervised* pattern* cognition* technique that may be used to identify patterns in data and build associated models. Then these models may be used for developing more complex classification systems with* supervised* pattern* recognition* techniques such as LDA, SIMCA, or ANN.

An essential factor influencing the efficiency of the predictive models is the quality of the data clustering in the score plots. The sensitivity and selectivity of the class assignment are strongly influenced by the relative position of the clusters and by their dispersion. The larger the distances between the clusters and the lower the cluster dispersion, the better the modeling and discrimination power of the PCA models. The quality of the data clustering may often be improved by signal preprocessing techniques [9, 17, 18]. However, the selection of the best preprocessing function is not always obvious, as often an improved sensitivity is obtained at the expense of the selectivity or vice versa [19]. For example, increased distances between the clusters are often generated by signal preprocessing functions that are also increasing the dispersion of the clusters. In addition, the positive effects of different preprocessing functions are often compared in a qualitative and thus relatively subjective manner, that is, by the visual inspection of the associated PCA score plots.

In this paper, we are proposing a set of indicators inspired from analytical geometry that may be used for an objective quantitative assessment of the data clustering quality in the PCA score plots, as well as a global clustering quality coefficient (GCQC) that is a measure of the overall efficiency of the PCA class assignment. The use of these indicators is illustrated by a comparative study performed for the identification of the preprocessing function that is generating the most efficient PCA system screening for amphetamines based on their GC-FTIR spectra. The effect of three feature weights on the clustering quality is discussed in comparison with the cluster relative positions and dispersions obtained in the case of unprocessed spectra. The ranking of the feature weights indicated by the GCQC is explained based on the dynamics of the cumulated explained variance and on the estimated density distributions calculated with the PC1 and PC2 scores associated with stimulant amphetamines (class M), hallucinogenic amphetamines (class T), and negatives (class N). The hierarchy obtained with the GCQC was validated by using quadratic discriminant analysis (QDA) [20].

Amphetamines are the main recreational drugs of abuse and thus important efforts are made to develop chemometrical tools allowing the automated detection of these drugs [21]. In order to improve the celerity of forensic procedures, these systems must be frequently updated, as new analogues are constantly emerging on the black market. On the other hand, taking into account the legal implications of the detection results, the expectations regarding the sensitivity and selectivity of these systems are very demanding.

**Figure 1:**
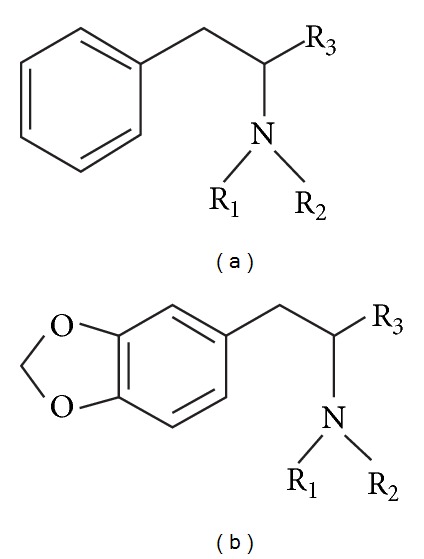
Molecular structures of the main amphetamine analogues: (a) stimulant amphetamines (amphetamine analogues, class M) and (b) hallucinogenic amphetamines (3,4-methylenedioxyamphetamine (MDA) analogues, class T).

Internationally, amphetamine is a Schedule II drug under the Convention on Psychotropic Substances [21, 22] in accordance with the Convention on Psychotropic Substances of 1971. Amphetamine analogues, often referred to as “amphetamines” or “substituted amphetamines,” contain amphetamine as a molecular skeleton. Substituted amphetamines, such as methamphetamine, whose basic molecular structure contains an aromatic ring linked by an aliphatic side chain to an amino group (see Figure 1(a)), are also stimulants [9, 14]. Other substituted amphetamines, such as 3,4-methylenedioxyamphetamine (MDA) and its analogues (see Figure 1(b)), are Schedule I drugs, as they have a pronounced hallucinogenic effect.

This study was performed in order to determine the GC-FTIR spectra preprocessing function yielding the best PCA clustering quality and associated models for performing an automated detection of amphetamines. The effect of three different preprocessing functions was assessed by an objective clusters analysis, based on analytical geometry indicators and GCQC. The results indicate that all three preprocessing functions increase the efficiency of the detection system. Although no information about the biological activity or toxicity of amphetamines was introduced in the input database, the illicit amphetamines may be detected according to their primary (stimulant or hallucinogenic) biological activity. This result is explained by the correlation between the biological effect of amphetamines and the substitution pattern of the aromatic ring present in their molecular skeleton, the information about the latter structural feature being maintained by the GC-FTIR spectra. GCQC has indicated which spectra preprocessing function is the most fit for purpose, that generates the PCA models with the best predictive (modeling and discriminating) power.

## 2. Experimental Part

The PCA training set consists of 30 GC-FTIR spectra, seven of which are the main illicit stimulant amphetamines. These amphetamine analogues have been assigned the class code M, as they all have in their molecular structure a monosubstituted aromatic ring (see Figure 1). The training set also contains the main hallucinogenic amphetamines. This group is formed by six 3,4-methylenedioxyamphetamine (MDA) analogues and has been assigned the code class T, as the molecular skeleton of all these compounds contains a trisubstituted aromatic ring (see Figure 1). The rest are spectra of nonamphetamines, representing compounds of toxicological interest (class code N). The experimental conditions in which the spectra have been recorded are presented in detail in previous studies [9, 14]. The analyzed spectra were recorded between 4000 and 600 cm^−1^, and the absorbance was measured every 5 cm^−1^. Thus, each spectrum is a vector with 681 variables.

The spectra in the database have been divided into two distinct classes, class I including the positives (M and T) and class II the negatives (N). The effect of three spectra preprocessing functions *w*, *w* ^2^, and (*w*−1)^2^ on the efficiency of the PCA based amphetamines screening system was evaluated in comparison with the results obtained with unprocessed spectra. The form of the feature weight *w* is given by [9]

(1) $$\begin{array}{c} w_{k}=\frac{\sum \left(A_{\text{I}}^{2}/N_{\text{I}}\right)+\sum \left(A_{\text{II}}^{2}/N_{\text{II}}\right)-2\sum \sum \left(A_{\text{I}}A_{\text{II}}/N_{\text{I}}N_{\text{II}}\right)}{\sum \left({\left(A_{\text{I}}-\bar{A_{\text{I}}}\right)}^{2}/N_{\text{I}}\right)+\sum \left({\left(A_{\text{II}}-\bar{A_{\text{II}}}\right)}^{2}/N_{\text{II}}\right)}, \end{array}$$

where *A*_I_ and *A*_II_ are the absorptions corresponding to the samples of classes I and II, and *N*_I_ and *N*_II_ are the numbers of samples in classes I and II. The functions *w* and *w* ^2^ act as selective amplifiers; that is, they enhance the intensity of the absorptions according to their modeling and/or discrimination power (*w* ^2^ > *w* > 1) and leave unchanged the absorptions corresponding to irrelevant wave numbers (*w* = 1). The function (*w*−1)^2^ acts as an amplifying selector, amplifying the absorptions with an important modeling and/or discrimination power and cancelling the absorptions recorded at irrelevant wave numbers, for which (*w*−1)^2^ = 0 [9].

PCA was performed by using the* MATLAB 2012a* software. The optimum number of PCs was established by taking into account the dynamics of the cumulated explained variance. The score plots revealed the existence of three clusters, formed by the class of stimulant amphetamines (class M), hallucinogenic amphetamines (class T), and negatives (class N). The best clustering results were obtained in all cases with the PC1 versus PC2 score plot.

A* MATLAB 2012a* application was built in order to assess quantitatively the relative position and the density of the clusters. As the results have indicated that each spectra preprocessing function has other advantages, a GCQC characterizing the overall predictive power of the PCA based detection system was defined. This global coefficient was also included in the* MATLAB 2012a* application and used to obtain an objective ranking of the spectra preprocessing functions from the point of view of their positive effect on the quality of the clusters in comparison with the results obtained with unprocessed spectra.

The results have been explained based on density distributions determined with a normal kernel estimator. The feature weight hierarchy obtained with the GCQC was validated by using a supervised pattern recognition technique, that is, QDA.

## 3. Results and Discussion

### 3.1. Detecting Amphetamines with Principal Component Analysis

The system built for the detection of stimulant M and hallucinogenic T amphetamines is based on PCA. The assignment of the class of an unknown occurs depending on the cluster where the point associated with this compound falls in the score plots.

**Figure 2:**
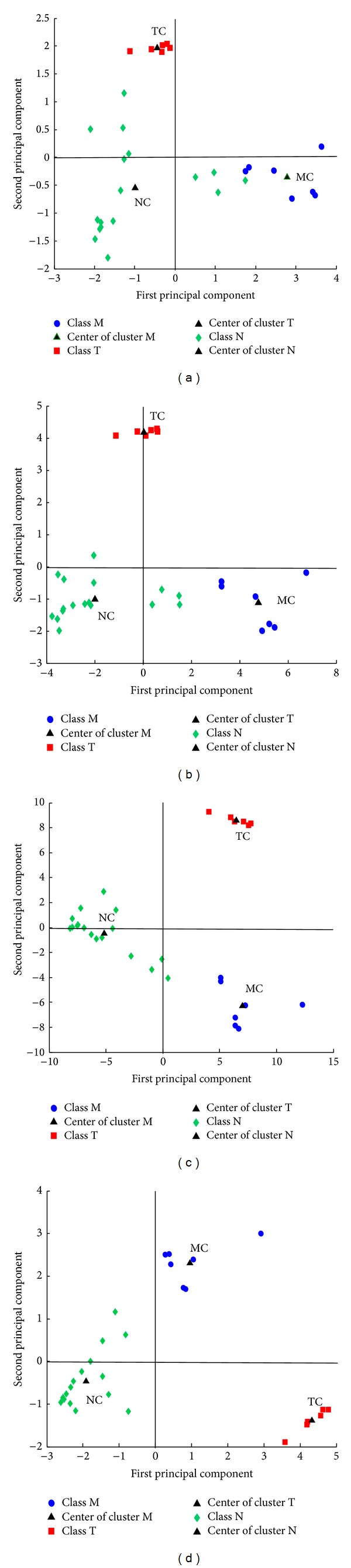
PC1 versus PC2 score plots associated with the GC-FTIR spectra of stimulant amphetamines (class M), hallucinogenic amphetamines (class T), and negatives (class N) for: (a) unprocessed spectra, (b) spectra preprocessed with the *w* selective amplifier, (c) spectra preprocessed with the *w* ^2^ selective amplifier, and (d) spectra preprocessed with the (*w*−1)^2^ amplifying selector. MC—center of the M cluster, TC—center of the T cluster, and NC—center of the N cluster.

The predictive power of the detection system may be improved by preprocessing the GC-FTIR spectra with various functions that may yield better defined clusters (see Figure 2). The more distant the clusters (M, T, N) are from each other in the *n*-dimensional space defined by the principal components (PC), the more selective the detection method is, that is, the smaller the probability of obtaining the false positives (M or T) or false negatives (N) is. At the same time, the more dense each cluster is, the better the sensitivity of the detection method is, that is, the larger the probability of correct classifications (true positive and true negative) is.

### 3.2. Assessing the Clustering Quality with Quantitative Indicators Derived from Analytical Geometry

The quality of the clusters found in the PCA score plots is usually assessed by visual inspection. This is a relatively subjective method, which can be easily misleading in assessing the overall improvement of the predictive power of the system. In order to obtain a more objective evaluation and overall comparison of the detection performances yielded by each feature weight, we have used a set of quantitative indicators derived from analytical geometry.

First, the center (*x*_*c*_, *y*_*c*_) of each cluster [19, 23] was determined:

(2) xc=1n∑i=1nxiyc=1n∑i=1nyi,

where *n* is the number of compounds forming the cluster, *x*_*i*_ is the score associated with compound *i* on the PC represented on the abscissa, and *y*_*i*_ is the score of the same compound on the PC represented on the ordinate.

Then the relative position of the clusters was characterized by using the following indicators:

(a) distance *D*_*i*,*i*′_ between two neighboring clusters, determined as the Euclidean distance [24] between the centers of clusters *i* and *i*′:
  (3) $$\begin{array}{c} D_{ii^{\prime }}=\sqrt{\overset{n}{\underset{j=1}{\sum }}{\left(x_{ij}-x_{i^{\prime }j}\right)}^{2}}, \end{array}$$
  and, in our case, *n* = 2, as the distance was determined for bidimensional representations (score plots);
(b) perimeter *P* of the polygon (triangle in our case) defined by the centers of the clusters;
(c) distance *A*_*i*,*i*′_ between the closest two points on the periphery of two different clusters *i* and *i*′.

The larger *D*_*i*,*i*′_, *P*, and *A*_*i*,*i*′_ are, the better the cluster discrimination is. In our case, these indicators confirm that all the tested spectra preprocessing functions are improving the dissociation of the clusters. Perimeter *P* is considerably higher in these cases than in the case of unprocessed spectra (see Table 1). The largest *D*_*i*,*i*′_, *P*, and *A*_*i*,*i*′_ are generated by the *w* ^2^ selective amplifier.

However, the efficiency of the detection system is influenced not only by the distance between the centers of clusters but also by the dispersion of the clusters. The effect of cluster condensation generated by each preprocessing function was assessed by using the following indicators:

- radius *R* of a cluster, calculated as the distance between the center and most peripheral point of the cluster;
- distance *L* between the two most distant points on the periphery of the same cluster;
- dispersion *D* of the points forming a cluster, calculated as the mean Euclidean distance between the center and the component points of a cluster.

These indicators characterize the density of a cluster. In other words, the lower *R*, *L*, and *D* are, the higher its density is. Tables 3 and 4 show that the *w* ^2^ selective amplifier, which generates the best results from the point of view of the relative position of the clusters, yields in the same time the most important cluster dispersion, that is, the largest cluster radii *R*, distances *L* between the most distant peripheral points, and dispersion *D* of all three clusters (M, T, and N). The best results from the point of view of the cluster density are obtained with the amplifying selector (*w*−1)^2^ (see Table 4). On the other hand, this feature weight has only a moderate positive effect from the point of view of the relative position of the clusters (see Table 1).

### 3.3. Assessing the Global Efficiency of the PCA Class Assignment

In conclusion, each preprocessing function has other advantages. Therefore, we have defined a global clustering quality coefficient (GCQC) that is assessing the overall predictive power of the PCA models. In our particular case, it is used for evaluating the overall effect of each preprocessing function on the PCA detection efficiency. The GCQC is defined based on the indicators previously used to characterize the relative position and the dispersion of the clusters; that is,

(4) $$\begin{array}{c} \text{CGQC}=\sqrt[{5}]{\frac{1}{\sum R_{i}}\cdot \frac{1}{\sum D_{i}}\cdot \frac{1}{\sum L_{i}}\cdot \sum A_{ii^{\prime }}\cdot P}, \end{array}$$

where *R*_*i*_ is the radius of cluster *i*, *D*_*i*_ is the dispersion of cluster *i*, *L*_*i*_ is the distance between the two most distant points of cluster *i*, *A*_*ii*′_ is the distance between the two closest peripheral points belonging to the neighboring clusters *i* and *i*′, and *P* is the perimeter.

Table 5 confirms that all the feature weights that were used for spectra preprocessing are improving the clustering quality. The largest GCQC is obtained for the (*w*−1)^2^ amplifying selector, which is ensuring the best global performances, that is, the best selectivity and sensitivity in detecting amphetamines. The *w* and *w* ^2^ selective amplifiers lead to smaller, almost equal GCQCs (see Table 5), indicating that their advantage of generating more distant clusters is offset by the disadvantage of yielding more dispersed clusters.

A first explanation for these results is given by the number of PCs found adequate to perform PCA in each case.  Table 6 shows the dynamics of the cumulated explained variance obtained for each feature weight. A number of 10 PCs were necessary to compress the useful information in the case of unprocessed spectra and of the *w* preprocessed spectra. However, the cumulated explained variance obtained for *w* preprocessed spectra is larger (95.9%) than for unprocessed spectra (93.8%). Only 7 PCs were necessary in the case of *w* ^2^ preprocessed spectra which are associated with nearly the same cumulated explained variance than in the case of *w* preprocessed spectra, although slightly smaller (95.7%). On the other hand, the first 3 PCs were enough to explain a cumulated explained variance of 95.8% in the case of (*w*−1)^2^ preprocessed spectra. The results are consistent with the feature weight hierarchy indicated by the GCQC (see Table 5).

We have mentioned before that the PC2 versus PC1 score plot yielded the best clustering in all cases.  Table 6 also shows that the cumulated explained variance of the first two PCs is much larger in the case of (*w*−1)^2^ preprocessed spectra (92.8%) than in the rest of the cases.

### 3.4. Assessing the Predictive Power of the PCA Detection System

In order to explain the results obtained with the GCQC and the associated analytical geometry indicators, each cluster has been analyzed by using the estimated density distribution of the PCA scores associated with GC-FTIR spectra. The cluster density was determined with a normal kernel estimator evaluated at 100 equidistant points *x*_*i*_ that cover the whole range of PCA scores determined for each class of modeled compounds [25–27]:

(5) $$\begin{array}{c} f\left(x\right)=\frac{1}{nh}\overset{n}{\underset{i=1}{\sum }}K\left(\frac{x-x_{i}}{h}\right), \end{array}$$

where *h* = 0.94 is the smoothing leveling parameter, *n* = 100, and *K* is the kernel function.

**Figure 3:**
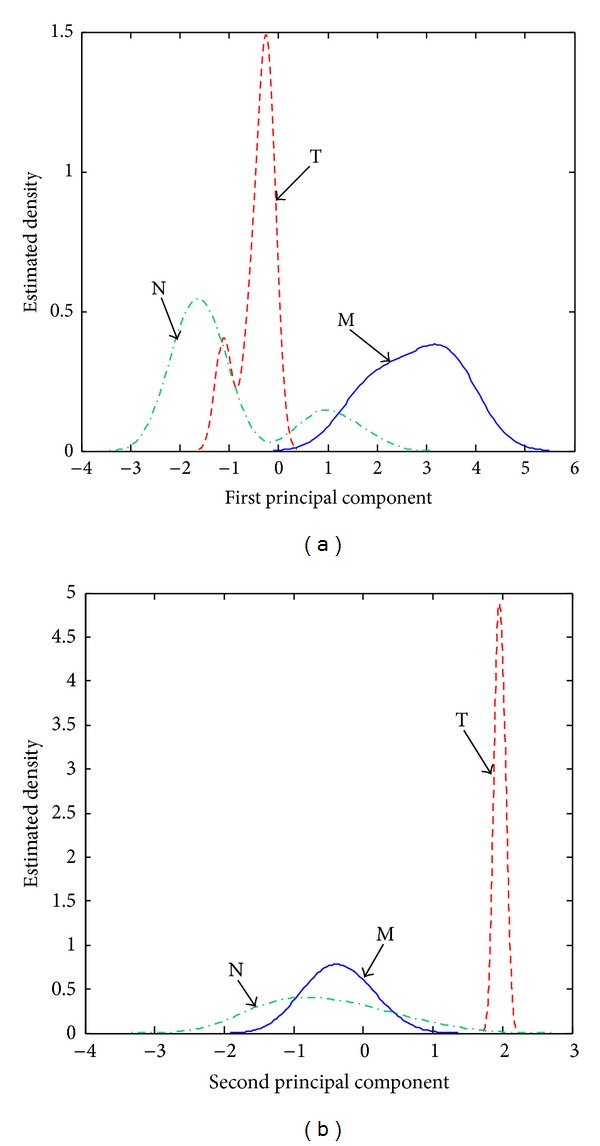
Estimated density distributions calculated with the (a) PC1 scores and (b) PC2 scores associated with stimulant amphetamines (class M), hallucinogens (class T), and negatives (class N) in the case of unprocessed spectra.

The estimated density distributions determined for unprocessed spectra indicate that an efficient detection can be obtained only for hallucinogens, especially due to the large positive PC2 scores (see Figure 3). Stimulant amphetamines and negatives can be distinguished better based on their PC1 scores. However, the negatives characterized by relatively large positive PC1 scores will most probably be classified as (false) M positives and stimulant amphetamines characterized by relatively small positive PC1 scores will be classified as (false) negatives N.

**Figure 4:**
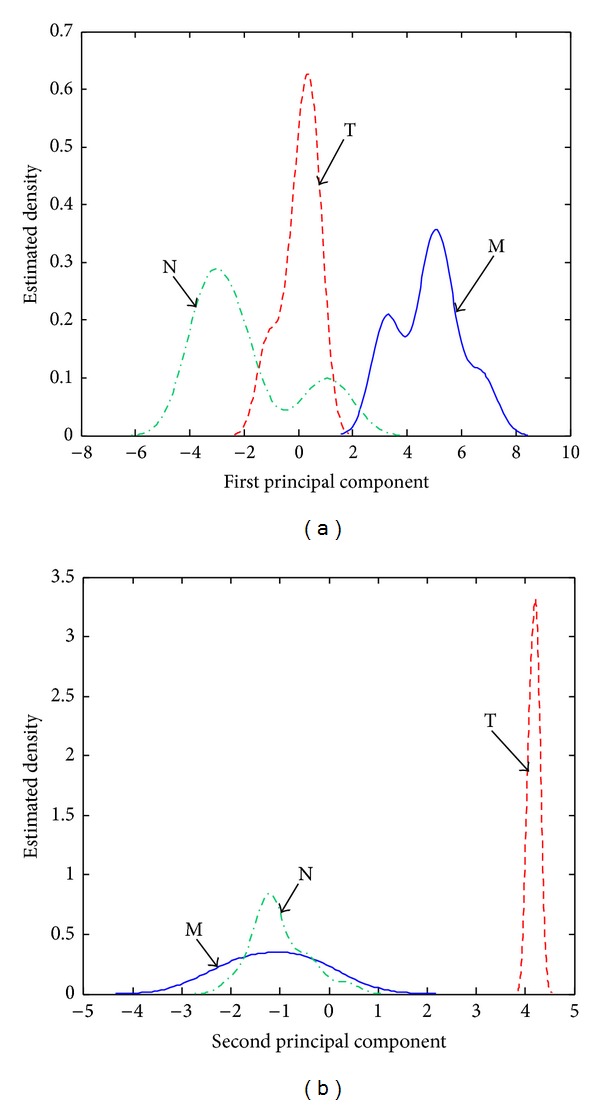
Estimated density distributions calculated with the (a) PC1 scores and (b) PC2 scores associated with stimulant amphetamines (class M), hallucinogens (class T), and negatives (class N) in the case of spectra preprocessed with the *w* selective amplifier.

The GCQC increases with 10% in the case of the spectra preprocessed with the *w* selective amplifier in comparison with the case of unprocessed spectra. The estimated density distributions determined for this feature weight (see Figure 4) show that its main effect on the relative position of the clusters is an improved detection of the hallucinogens (T). The larger distances *D*_*i*,*i*_ between the centers of the M, T, and N clusters and perimeter (see Table 1) and the increased distances *A*_*i*,*j*_ between the two closest points on the periphery of two of the M, T, and N clusters (see Table 2) are contributing especially to the improvement of the sensitivity and selectivity of the T cluster (see Figure 4(b)). The overlap of the M and N estimated density distributions determined with the associated PC1 scores is less important than in the case of the unprocessed spectra. However, false M and N classifications may still be expected due to significant cluster dispersion.

**Figure 5:**
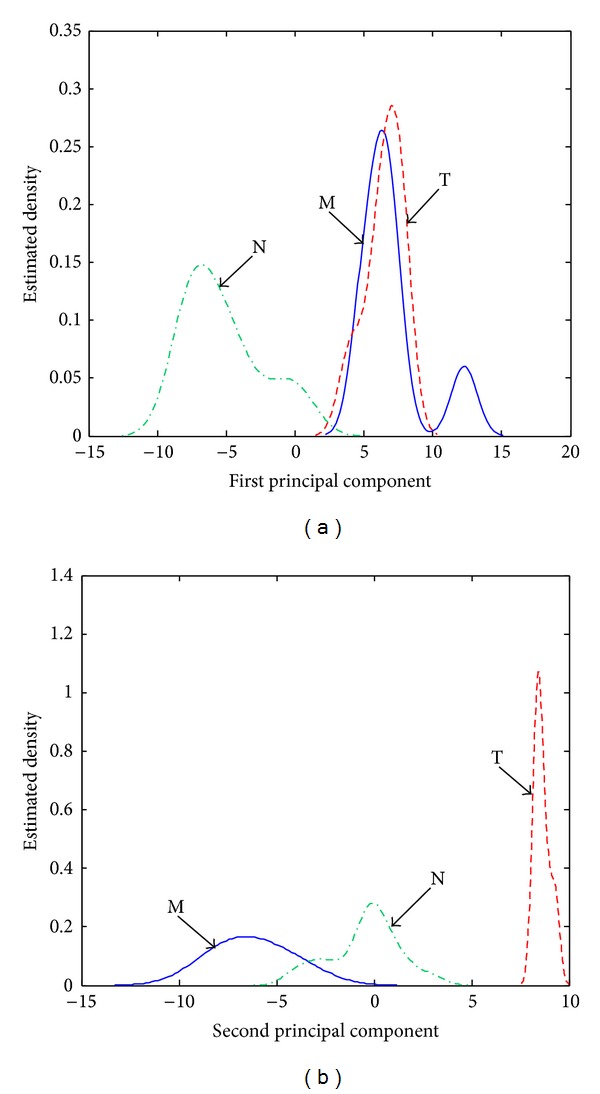
Estimated density distributions calculated with the (a) PC1 scores and (b) PC2 scores associated with stimulant amphetamines (class M), hallucinogens (class T), and negatives (class N) in the case of spectra preprocessed with the *w* ^2^ selective amplifier.

The estimated density distributions determined for *w* ^2^ show that the hallucinogens form the best defined cluster (see Figure 5). Similarly to the case of the *w* selective amplifier, these compounds can be distinguished from the other substances due to their high positive PC2 scores. The *w* ^2^ selective amplifier generates the largest distances characterizing the relative positions of the clusters. As a result, the M and N estimated density distributions determined for PC2 scores are overlapping less than in the case of the *w* selective amplifier (see Figure 4(b)) or of the unprocessed spectra (see Figure 3(b)). However, *w* ^2^ also generates the largest cluster dispersion. In this case, the dispersion is so important that it counterbalances the positive effect related to cluster relative positions stronger than in the case of the *w* selective amplifier. The GCQC is sensitive to these variations. As Table 5 shows, the GCQC obtained for *w* ^2^ represents only 95.43% of the value determined for the *w* selective amplifier.

**Figure 6:**
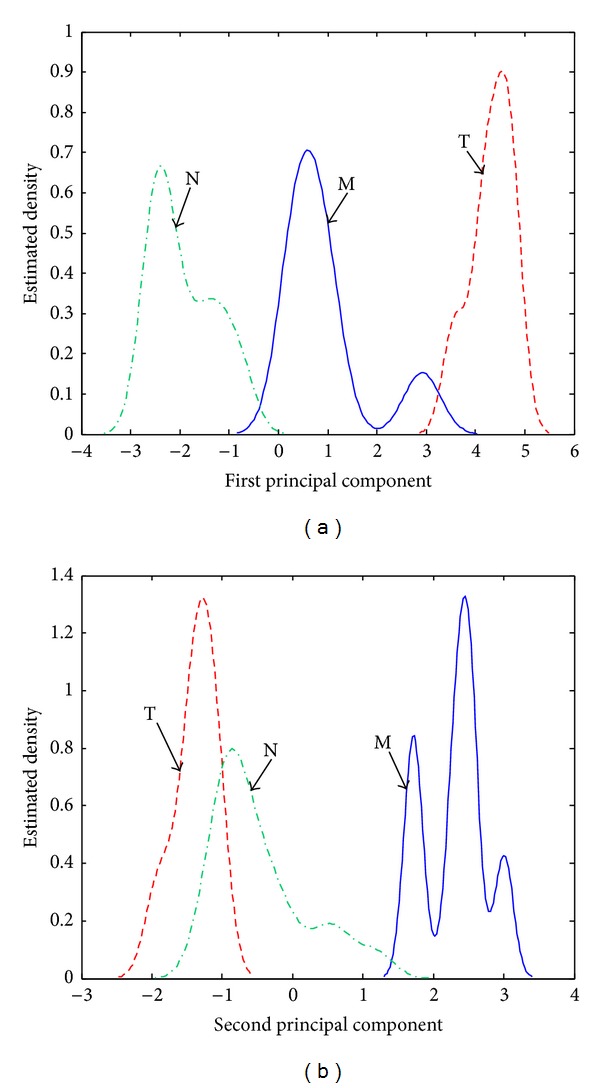
Estimated density distributions calculated with the (a) PC1 scores and (b) PC2 scores associated with stimulant amphetamines (class M), hallucinogens (class T), and negatives (class N) in the case of spectra preprocessed with the (*w*−1)^2^ amplifying selector.

The estimated density distributions (see Figure 6) indicate that the (*w*−1)^2^ amplifying selector leads to the best discrimination of amphetamines according to their biological activity and associated toxicity. The most important positive effect of this feature weight in comparison with the *w* and *w* ^2^ selective amplifiers is the improved discrimination of the clusters of stimulant amphetamines (class M) and of negative compounds (class N). Indeed, the cluster of stimulant amphetamines is found in quadrant I, the cluster of negatives in quadrants II and III, and the cluster of hallucinogenic amphetamines in quadrant IV of the PC2 versus PC1 score plot (see Figure 2(d)).

### 3.5. Validation of the Global Clustering Quality Coefficient (GCQC)

In order to validate the GCQC based ranking of the spectra preprocessing functions according to the predictive power of the PCA models they are generating, a validation set formed by the PC1 and PC2 scores associated with the GC-FTIR spectra of 159 compounds of forensic interest was subjected to QDA [20, 28]. These compounds represent stimulant amphetamine analogues, homologues, and derivatives (e.g., 2- and 1-phenethylamines); hallucinogenic amphetamines (3,4-methylenedioxy and 2,5-dimethoxyamphetamine analogues and derivatives, 3,4,5-trimethoxyamphetamine); their main precursors (e.g., sympathomimetic amines such as ephedrine, its analogues and isomers, safrole, isosafrole, PMK, BMK, etc.); compounds structurally similar to the side chain of amphetamines (e.g., putrescine and cadaverine); and other controlled substances (cocaine, opiates, opioids, etc.).

**Figure 7:**
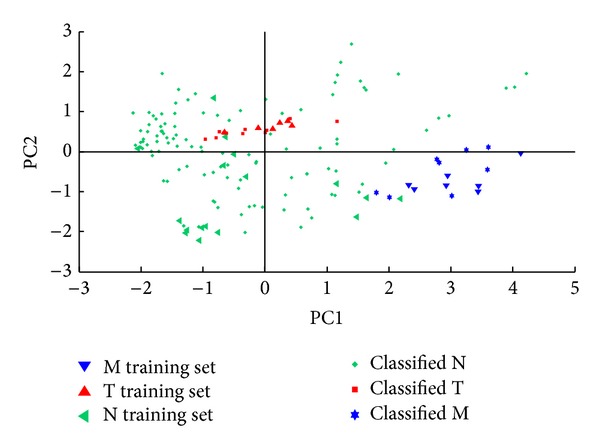
Quadratic discriminant analysis performed with the (PC1, PC2) scores associated with stimulant amphetamines (class M), hallucinogens (class T), and negatives (class N) in the case of unprocessed spectra.

**Figure 8:**
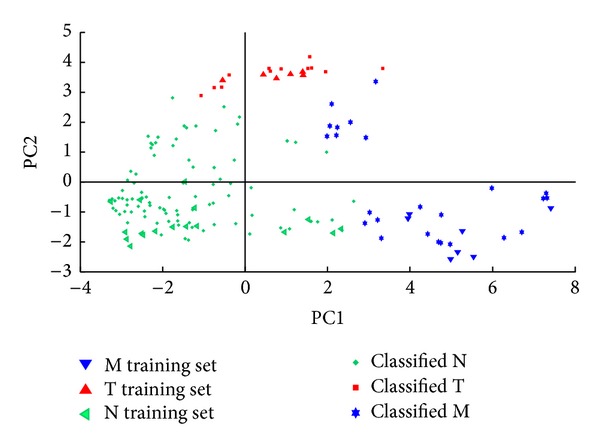
Quadratic discriminant analysis performed with the (PC1, PC2) scores associated with stimulant amphetamines (class M), hallucinogens (class T), and negatives (class N) in the case of spectra preprocessed with the *w* selective amplifier.

**Figure 9:**
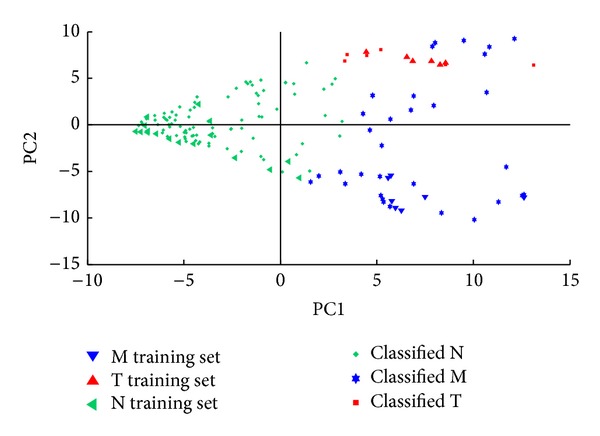
Quadratic discriminant analysis performed with the (PC1, PC2) scores associated with stimulant amphetamines (class M), hallucinogens (class T), and negatives (class N) in the case of spectra preprocessed with the *w* ^2^ selective amplifier.

**Figure 10:**
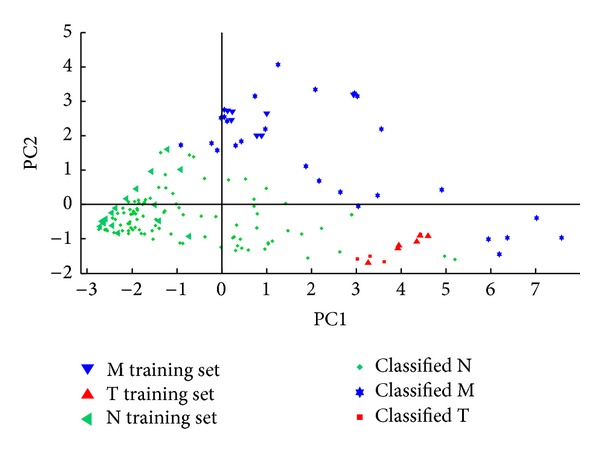
Quadratic discriminant analysis performed with the (PC1, PC2) scores associated with stimulant amphetamines (class M), hallucinogens (class T), and negatives (class N) in the case of spectra preprocessed with the (*w*−1)^2^ amplifying selector.

QDA is a supervised pattern recognition technique closely related to LDA, where it is assumed that the measurements from each class are normally distributed. The quadratic version of discriminant analysis was preferred as, unlike LDA, in QDA there is no assumption that the covariance of each of the classes is identical. The classification results are displayed in Figures 7, 8, 9, and 10.

The classification results obtained with QDA are synthesized in Table 7. The classification rate indicates the percentage of compounds that could be classified (correctly or not) by the system. Among these, the correct classification rate indicates the percentage of compounds that have been classified as true positives (amphetamines classified as such) and true negatives (nonamphetamines classified as such). Unlike the case of unprocessed spectra, all the class assignments performed with preprocessed spectra yield classification rates of 100%. The correct classification rates confirm the ranking provided by GCQC for the spectra preprocessing functions and their positive effect on the quality of the clusters in comparison with the case of unprocessed spectra (see Tables 5 and 7). The correct classification rates determined for each PCA model (M, T, and N) are in accordance with the results indicated by the estimated density distributions.

The increased predictive power indicated by the GCQC in the case of the *w* selective amplifier is validated by an improved correct classification rate. As the estimated density distributions have indicated, this positive effect is due to the improvement of the sensitivity and selectivity of the clusters formed by hallucinogenic and stimulant amphetamines, the T and M correct classification rates reaching 100% and 95.23%, respectively. However, these results are obtained at the expense of the sensitivity of the N model.

The GCQC has indicated a slightly lower predictive power for the models generated by spectra preprocessing with the *w* ^2^ selective amplifier. Indeed, the correct classification rate becomes 84.90% in this case. As the estimated density distributions have indicated, this is due to the large cluster dispersion that affects the sensitivity of the N model even more than in the case of the *w* selective amplifier.

Finally, the GCQC has forecasted that the best results may be obtained with the spectra preprocessed with the (*w*−1)^2^ amplifying selector. This function generates the best balance between the relative position and the dispersion of each cluster. As a result, the correct classification rate reaches 89.93%. Consistent with the information provided by the estimated density distributions, the most important positive effect of the (*w*−1)^2^ amplifying selector in comparison with the second best preprocessing function, that is, the *w* selective amplifier, is related to an improved sensitivity of the M and N models. With this feature weight, no false negatives have been recorded. Taking into account the purpose of the screening system, its most important requirement is related to the sensitivity of the M and T models. The only acceptable compromise is related to the selectivity of these models: false positives are not desirable, but acceptable.

## 4. Conclusions

The literature presents many cases in which the natural clustering of the data has been improved by preprocessing the analytical signal used for characterizing the target compounds. However, an improved selectivity is often obtained at the expense of sensitivity of the PCA class assignment, and often the feature weight yielding the most efficient screening system is selected subjectively.

We have proposed a set of quantitative indicators inspired from analytical geometry that may be used for characterizing objectively the sensitivity, selectivity, and overall efficiency of unsupervised pattern recognition applications. Their use has been illustrated by a comparative study on the effect of three spectra preprocessing functions on the efficiency of a PCA based screening system designed to assign the class of amphetamines according to the type of their biological activity.

Analytical geometry indicators have been used for an objective assessment of the cluster relative position and of the cluster density, which are directly influencing the predictive power of the PCA models, and thus the sensitivity and selectivity of the detection system. The results show that the preprocessing function that is yielding the best cluster relative position is not necessarily ensuring the best efficiency in class assignment. For example, the *w* ^2^ selective amplifier generates an important cluster dispersion, which shadows its positive effect of yielding the largest distances between the clusters.

In order to obtain an objective ranking, we have defined a GCQC that allows the quantitative overall assessment of the efficiency of the PCA based screening systems. The results indicate that the best detection of amphetamines according to their biological activity is obtained when the GC-FTIR spectra are preprocessed with the (*w*−1)^2^ amplifying selector. Although this spectra preprocessing function yields only a moderate improvement of the cluster relative position, it yields the class assignment because it ensures the best balance between the distances between the clusters and their dispersion.

The hierarchy indicated by the GCQC has been explained by analyzing the dynamics of the cumulated explained variance, the associated number of PCs, and the estimated density distributions determined for the PC1 and PC2 scores associated with stimulant amphetamines, hallucinogenic amphetamines, and negative compounds. The results indicate that the (*w*−1)^2^ amplifying selector maximizes the sensitivity of the detection system.

In conclusion, GCQC may be used to compare score plots provided by this nonhierarchical clustering technique in the same way as the cophenetic correlation coefficient may be used for ranking different algorithms generating hierarchical classification trees (dendrograms). The same indicators may be used for deciding the size and nature of the training set. In other words, they can help us make objective evaluations for deciding matters such as what is the best number and nature of samples to include in the training set? Should we keep in the training set samples that are not outliers but are found on the border of a given cluster? How many and which variables may be eliminated without affecting the efficiency of the class assignment? How many models should be built? Another important advantage is that this approach saves the time spent for evaluating the predictive power of the models by more complex classification techniques.

Last but not least, we would like to emphasize that the use of quantitative (analytical geometry and GCQC) indicators presents the major advantage of allowing the automatic evaluation of the cluster relative position and dispersion, as well as of the overall predictive power of the models. The described procedure has been developed as a* MATLAB* algorithm for the overall assessment of PCA based screening systems.

## Figures and Tables

**Table 1:** Distance *D*_*i*,*i*′_ between the centers of the M, T, and N clusters and perimeter.

Cluster	M	T	N	Perimeter (*P*)
Scores associated with the unprocessed spectra				
M	—	3,9721	3,7719	10,3115
T	3,9721	—	2,5675	
N	3,7719	2,5675	—	

Scores associated with the spectra preprocessed with the *w* selective amplifier				
M	—	7,1051	6,7704	19,4517
T	7,1051	—	5,5762	
N	6,7704	5,5762	—	

Scores associated with the spectra preprocessed with the *w* ^2^ selective amplifier				
M	—	14,8991	13,4897	43,1363
T	14,8991	—	14,7475	
N	13,4897	14,7475	—	

Scores associated with the spectra preprocessed with the (*w*−1)^2^ amplifying selector				
M	—	5,0030	3,9815	15,2936
T	5,0030	—	6,3091	
N	3,9815	6,3091	—	

**Table 2:** Distance *A*_*i*,*i*′_ between the two closest points on the periphery of two of the M, T, and N clusters.

Cluster	M	T	N
Associated with the unprocessed spectra			
M	—	2,9038	0,1687
T	2,9038	—	0,7674
N	0,1687	0,7674	—

Associated with the spectra processed with the *w* selective amplifier			
M	—	5,3510	1,7744
T	5,3510	—	3,8290
N	1,7744	3,8290	—

Associated with the spectra processed with the *w* ^2^ selective amplifier			
M	—	13,3527	4,6160
T	13,3527	—	12,5518
N	4,6160	12,5518	—

Associated with the spectra processed with the (*w*−1)^2^ amplifying selector			
M	—	4,4680	1,9256
T	4,4680	—	4,3929
N	1,9256	4,3929	—

**Table 3:** Cluster radius *R* and distance *L* between the two most distant points on the periphery of the same cluster for the M, T, and N clusters.

M		T		N	
*R*	*L*	*R*	*L*	*R*	*L*
Scores associated with unprocessed spectra					
1,0330	1,9269	0,6743	0,9912	2,7387	3,9615

Scores associated with spectra processed with *w* selective amplifier					
2,1889	3,5424	1,1811	1,7442	3,4997	5,2837

Scores associated with spectra processed with *w* ^2^ selective amplifier					
5,2805	7,5303	2,4913	3,8042	6,6759	9,6853

Scores associated with spectra processed with (*w*−1)^2^ amplifying selector					
2,0889	2,6859	0,8980	1,4180	1,8189	2,6080

**Table 4:** Dispersion *D* of the M, T, and N clusters.

Dispersion	M	T	N
Unprocessed spectra	0,7455	0,2775	1,3093
Spectra processed with *w* selective amplifier	1,1985	0,4973	1,5926
Spectra processed with *w* ^2^ selective amplifier	2,2853	1,0685	2,7851
Spectra processed with (*w*−1)^2^ amplifying selector	0,7545	0,3942	0,8029

**Table 5:** Global clustering quality coefficient (GCQC) assessing the overall efficiency of the PCA class assignment.

Unprocessed spectra	0,8889
Spectra processed with the *w* selective amplifier	0,9774
Spectra processed with the *w* ^2^ selective amplifier	0,9327
Spectra processed with the (*w*−1)^2^ amplifying selector	1,2124

**Table 6:** Cumulated explained variance.

Principal component	PC1 (%)	PC2 (%)	PC3 (%)	PC4 (%)	PC5 (%)	PC6 (%)	PC7 (%)	PC8 (%)	PC9 (%)	PC10 (%)
Unprocessed spectra	37.9	**53.1**	63.3	71.8	78.4	82.9	87.0	89.8	92.1	**93.8**
*w* preprocessed spectra	44.6	**65.9**	73.6	79.3	84.6	89.0	91.6	93.4	94.8	**95.9**
*w* ^2^ preprocessed spectra	48.5	**80.0**	85.4	88.7	92.0	93.8	**95.7**	—	—	—
(*w*−1)^2^ preprocessed spectra	70.5	**92.8**	**95.8**	—	—	—	—	—	—	—

**Table 7:** Predictive power assessed by quadratic discriminant analysis (QDA).

	Classification rate (%)	Correct classification rate (%)	M correct classification rate (%)	T correct classification rate (%)	N correct classification rate (%)
Unprocessed spectra	94.12	**84.27**	66.66	85.71	87.02
*w* preprocessed spectra	100	**85.53**	95.23	100	83.20
*w* ^2^ preprocessed spectra	100	**84.90**	100	100	81.67
(*w*−1)^2^ preprocessed spectra	100	**89.93**	100	100	87.78
